# Supplementary material for: Gill Junction Injury and Microbial Disorders Induced by Microcystin-Leucine Arginine in Lithobates catesbeianus Tadpoles
Source: Toxins (Basel). 2022 Jul 13;14(7):479. doi: 10.3390/toxins14070479 (PMC9322459; doi:10.3390/toxins14070479)
Supplement: Supplementary file 1 [file toxins-14-00479-s001.zip › supplementary figure-done.pdf]

toxins

MDPI

Article

Gill Junction Injury and Microbial Disorders Induced by Microcystin-Leucine Arginine in *Lithobates catesbeianus* Tadpoles

Huiling Jiang, Jun He, Hui Wang, Lingling Zheng, Xiaoran Wang, Huijuan Zhang, Hailong Wu, and Yilin Shu

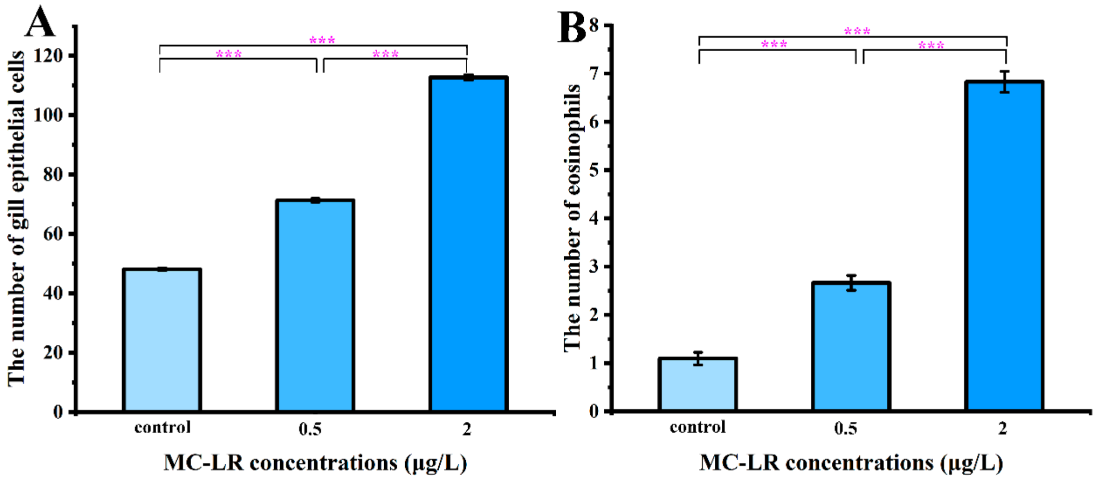

Figure S1. The number of in-ternal gill epithelial cells (A). The average number of eosinophils (B). All data were shown as mean ± SEM of six replicates (n = 6). * $p < 0.05$, ** $p < 0.01$, and *** $p < 0.001$ indicate significant difference. Abbreviations: EOS, Eosinophils. Gill filament epithelial cells are shown within the solid rectangle.

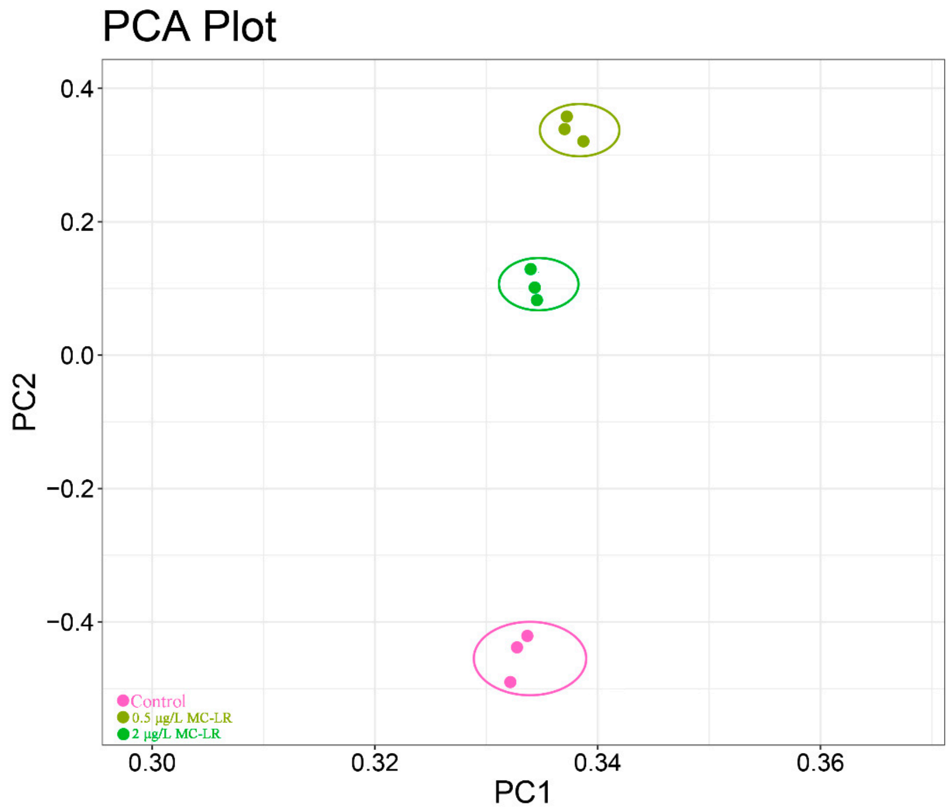

Figure S2. Principal component analysis of gene expression results for Control, 0.5, and 2 μg/L MC-LR group.

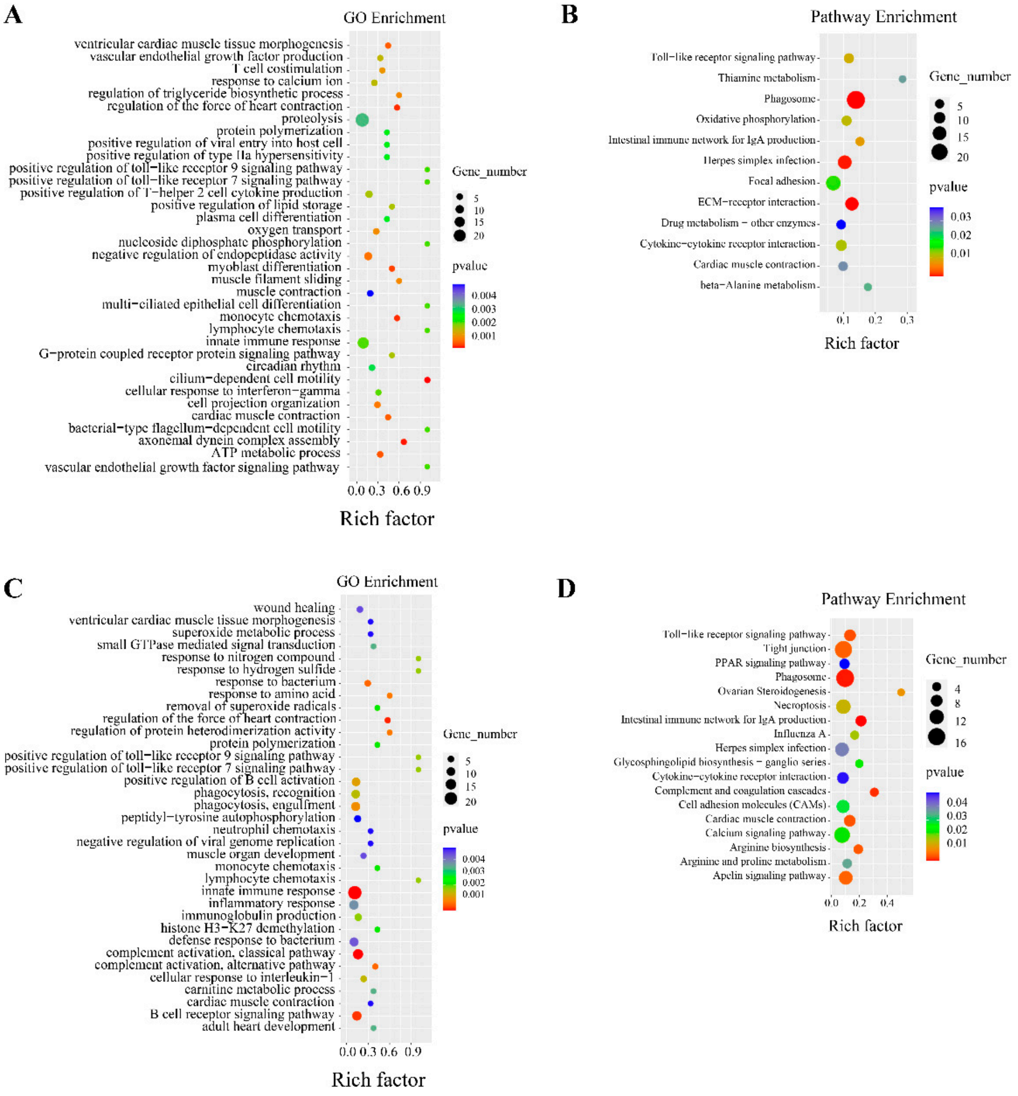

Figure S3. GO and KEGG enrichment analysis of DEGs. GO enrichment biological process at top 35 (A, C), KEGG pathways analysis of DEGs (B, D). 0.5 μg/L MC-LR group vs Control group (A, B). 2 μg/L MC-LR group vs Control group (C, D).

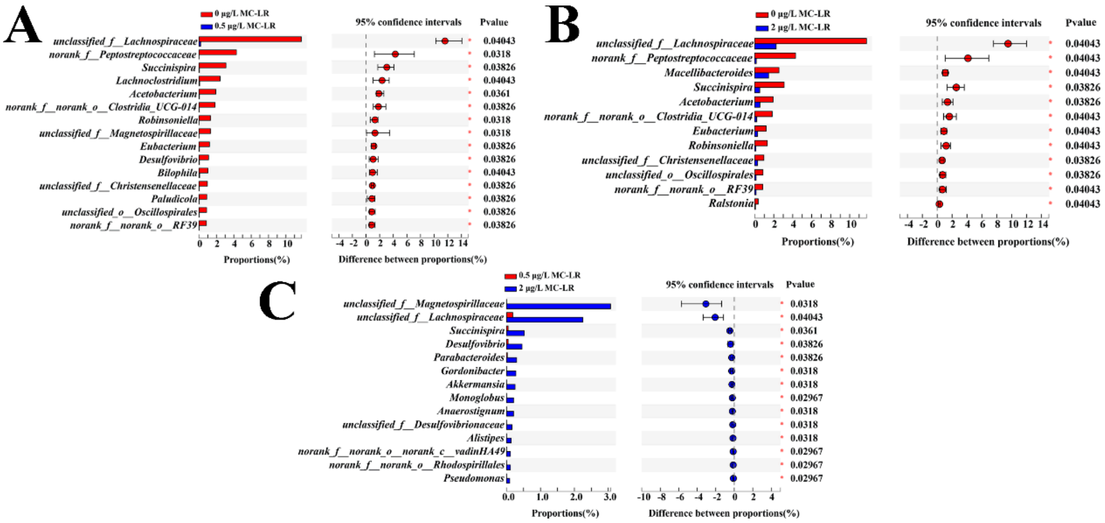

Figure S4. Gill microbiota remarkably different among MC-LR groups at genus level. Data present relative abundance (%) at genus level in each group by Wilcoxon signed rank test analysis. 0.5 μg/L MC-LR group vs Control group (A). 2 μg/L MC-LR group vs Control group (B). 0.5 μg/L MC-LR group vs 2 μg/L MC-LR group (C).
